# A reply to questions raised about FEV_1_Q and bronchodilator responsiveness

**DOI:** 10.1183/13993003.02025-2022

**Published:** 2023-01-19

**Authors:** Martin R. Miller, Sanja Stanojevic, David A. Kaminsky, Bruce R. Thompson

**Affiliations:** 1 Institute of Applied Health Research, University of Birmingham, Birmingham, UK; 2 Department of Community Health and Epidemiology, Dalhousie University, Halifax, NS, Canada; 3 Pulmonary Disease and Critical Care Medicine, University of Vermont Larner College of Medicine, Burlington, VT, USA; 4 Physiology Service, Department of Respiratory Medicine, The Alfred Hospital and School of Health Sciences, Swinburne University of Technology, Melbourne, Australia

## Abstract

We thank K. Rurak and H. Schotland for their feedback on the recent statements on assessing lung function changes over time in the European Respiratory Society (ERS)/American Thoracic Society (ATS) technical standard on pulmonary function test (PFT) interpretive strategies [1]. We accept that the section related to natural changes in lung function over time was limited and this was not ideal. For changes over time we could only comment on forced expiratory volume in 1 s (FEV_1_) as there is a lack of data for the other indices. This is a major gap in the literature that needs to be addressed.

## Abstract 2

**The recent ATS/ERS technical standard on pulmonary function test interpretive strategies is based on the available evidence** https://bit.ly/3hgQsm1

*Reply to K. Rurak and H. Schotland, and to T.P. Presti and D.C. Johnson*:

We thank K. Rurak and H. Schotland for their feedback on the recent statements on assessing lung function changes over time in the European Respiratory Society (ERS)/American Thoracic Society (ATS) technical standard on pulmonary function test (PFT) interpretive strategies [1]. We accept that the section related to natural changes in lung function over time was limited and this was not ideal. For changes over time we could only comment on forced expiratory volume in 1 s (FEV_1_) as there is a lack of data for the other indices. This is a major gap in the literature that needs to be addressed. While clinicians would like specific guidance that is easy to follow, the reality is that there is a great deal of uncertainty with using lung function to inform diagnosis and prognosis. Hitherto, guidance using percent of predicted has been the norm to interpret PFT measurements with little evidence to support this approach. The simplicity of using percent predicted comes at a cost of bias with respect to sex, age and size (*i.e.* height). Using FEV_1_ without reference to predicted values, either standardised by powers of height [2–4] or by the first centile value found in patients with abnormal lung function (FEV_1_Q) [5, 6], results in a grading scale of severity that is more closely associated with survival. Within the updated ERS/ATS technical standard on PFT interpretation we emphasise that the newer approaches proposed need to be tested against current practice to help improve decision making in the future. Existing evidence supports that FEV_1_Q accounts for differences in lung function decline between males and females [7] and so merits consideration. The incorporation of PFT measures into clinical guidelines was beyond the remit of the technical standard. Clinical guidelines supported with evidence may need to be revised in the future.

Separately, T.P. Presti and D.C. Johnson have raised concerns about the change in guidance on interpreting bronchodilator responsiveness (BDR). They correctly point out that moving from accepting a certain percentage change from baseline to a percentage of predicted value will change who is deemed to have a significant response. They imply that individuals who would have been deemed responsive by the old criteria but not by the new will be denied optimal treatment. The indications to perform a BDR test and the decision on treatment are separate issues. The revised BDR calculation addresses known biases implicit in calculating the percentage change of a starting (baseline) value. T.P. Presti and D.C. Johnson further state that percent of predicted is easier to understand than z-scores when considering severity of lung function impairment. Percent of predicted, although simpler, leads to biased interpretation, especially in older females. Continuing to use antiquated approaches because they appear to be simpler is a disservice to patients. Clinical decisions regarding diagnosis and treatment should be made based on a comprehensive assessment of medical history, physical examination, and all relevant data. Those data may include lung function, which should be expressed in a way that is impartial and evidence-based.

## Shareable PDF

10.1183/13993003.02025-2022.Shareable1 This one-page PDF can be shared freely online.
Shareable PDF ERJ-02025-2022.Shareable
