# Supplementary material for: A reply to questions raised about FEV1Q and bronchodilator responsiveness
Source: Eur Respir J. 2023 Jan 19;61(1):2202025. doi: 10.1183/13993003.02025-2022 (PMC9849703; doi:10.1183/13993003.02025-2022)
Supplement: Supplementary file 1 [file ERJ-02025-2022.Shareable.pdf]

EUROPEAN RESPIRATORY JOURNAL
CORRESPONDENCE
M.R. MILLER ET AL.

SHAREABLE PDF

A reply to questions raised about FEV_1Q and bronchodilator responsiveness

Martin R. Miller[1], Sanja Stanojevic[2], David A. Kaminsky[3] and Bruce R. Thompson[4]

[1]Institute of Applied Health Research, University of Birmingham, Birmingham, UK. [2]Department of Community Health and Epidemiology, Dalhousie University, Halifax, NS, Canada. [3]Pulmonary Disease and Critical Care Medicine, University of Vermont Larner College of Medicine, Burlington, VT, USA. [4]Physiology Service, Department of Respiratory Medicine, The Alfred Hospital and School of Health Sciences, Swinburne University of Technology, Melbourne, Australia.

Corresponding author: Martin R. Miller (mrmc2oho@gmail.com)

Check for updates

Shareable abstract (@ERSpublications)
The recent ATS/ERS technical standard on pulmonary function test interpretive strategies is based on the available evidence https://bit.ly/3hgQsm1

Cite this article as: Miller MR, Stanojevic S, Kaminsky DA, *et al*. A reply to questions raised about FEV_1Q and bronchodilator responsiveness. *Eur Respir J* 2023; 61: 2202025 [DOI: 10.1183/13993003.02025-2022].

This single-page version can be shared freely online.

Copyright ©The authors 2023.

This version is distributed under the terms of the Creative Commons Attribution Licence 4.0.

Received: 19 Oct 2022
Accepted: 7 Dec 2022

Reply to K. Rurak and H. Schotland, and to T.P. Presti and D.C. Johnson:

We thank K. Rurak and H. Schotland for their feedback on the recent statements on assessing lung function changes over time in the European Respiratory Society (ERS)/American Thoracic Society (ATS) technical standard on pulmonary function test (PFT) interpretive strategies [1]. We accept that the section related to natural changes in lung function over time was limited and this was not ideal. For changes over time we could only comment on forced expiratory volume in 1 s (FEV_1) as there is a lack of data for the other indices. This is a major gap in the literature that needs to be addressed. While clinicians would like specific guidance that is easy to follow, the reality is that there is a great deal of uncertainty with using lung function to inform diagnosis and prognosis. Hitherto, guidance using percent of predicted has been the norm to interpret PFT measurements with little evidence to support this approach. The simplicity of using percent predicted comes at a cost of bias with respect to sex, age and size (*i.e.* height). Using FEV_1 without reference to predicted values, either standardised by powers of height [2–4] or by the first centile value found in patients with abnormal lung function (FEV_1Q) [5, 6], results in a grading scale of severity that is more closely associated with survival. Within the updated ERS/ATS technical standard on PFT interpretation we emphasise that the newer approaches proposed need to be tested against current practice to help improve decision making in the future. Existing evidence supports that FEV_1Q accounts for differences in lung function decline between males and females [7] and so merits consideration. The incorporation of PFT measures into clinical guidelines was beyond the remit of the technical standard. Clinical guidelines supported with evidence may need to be revised in the future.
